# Prevalence, risk factors, and long-term outcomes of cerebral ischemia in hospitalized COVID-19 patients – study rationale and protocol of the CORONIS study: A multicentre prospective cohort study

**DOI:** 10.1177/23969873221092538

**Published:** 2022-04-06

**Authors:** Theresa J van Lith, Wouter M Sluis, Naomi T Wijers, Frederick JA Meijer, Karin Kamphuis-van Ulzen, Jeroen de Bresser, Jan Willem Dankbaar, Frederik MA van den Heuvel, M Louisa Antoni, Catharina M Mulders-Manders, Quirijn de Mast, Frank L van de Veerdonk, Frederikus A Klok, Anil M Tuladhar, Suzanne C Cannegieter, Marieke JH Wermer, H Bart van der Worp, Menno V Huisman, Frank-Erik de Leeuw

**Affiliations:** 1 Department of Neurology, Donders Center for Medical Neuroscience, Radboud University Medical Center, Nijmegen, The Netherlands; 2 Department of Neurology and Neurosurgery, Brain Center, University Medical Center Utrecht, Utrecht, The Netherlands; 3 Department of Neurology, Leiden University Medical Center, Leiden, The Netherlands; 4 Department of Medical Imaging, Radboud University Medical Center, Nijmegen, The Netherlands; 5 Department of Radiology, Leiden University Medical Center, Leiden, the Netherlands; 6 Department of Radiology and Nuclear Medicine, University Medical Center, Utrecht University, Utrecht, The Netherlands; 7 Department of Cardiology, Radboud University Medical Center, Nijmegen, The Netherlands; 8 Department of Cardiology, Heart and Lung Centre, Leiden University Medical Centre, Leiden, The Netherlands; 9 Department of Internal Medicine, Radboud University Nijmegen Medical Center, Nijmegen, The Netherland; 10 Department of Internal Medicine and Radboud Center for Infectious Diseases, Radboud University Medical Center, Nijmegen, The Netherlands; 11 Department of Medicine – Thrombosis and Hemostasis, Leiden University Medical Centre, Leiden, The Netherlands; 12 Department of Clinical Epidemiology, Leiden University Medical Centre, Leiden, The Netherlands

**Keywords:** COVID-19, SARS-CoV-2, silent cerebral ischemia, ischemic stroke

## Abstract

**Background:**

COVID-19 is often complicated by thrombo-embolic events including ischemic stroke. The underlying mechanisms of COVID-19-associated ischemic stroke, the incidence and risk factors of silent cerebral ischemia, and the long-term functional outcome in these patients are currently unknown.

**Patients and methods:**

CORONavirus and Ischemic Stroke (CORONIS) is a multicentre prospective cohort study investigating the prevalence, risk factors and long-term incidence of (silent) cerebral ischemia, and the long-term functional outcome among patients with COVID-19. We aim to include 200 adult patients hospitalized with COVID-19 without symptomatic ischemic stroke to investigate the prevalence of silent cerebral ischemia compared with 60 (matched) controls with MRI. In addition, we will identify potential risk factors and/or causes of cerebral ischemia in COVID-19 patients with (*n* = 70) or without symptomatic stroke (*n* = 200) by means of blood sampling, cardiac workup and brain MRI. We will measure functional outcome and cognitive function after 3 and 12 months with standardized questionnaires in all patients with COVID-19. Finally, the long-term incidence of (new) silent cerebral ischemia in patients with COVID-19 will be assessed with follow up MRI (*n* = 120).

**Summary:**

The CORONIS study is designed to add further insight into the prevalence, long-term incidence and risk factors of cerebral ischemia, and the long-term functional outcome in hospitalized adult patients with COVID-19.

## Introduction and rationale

The clinical course of coronavirus disease 2019 (COVID-19) is complicated by a high risk of thrombo-embolic complications, with a higher incidence in patients admitted to the intensive care unit (ICU) compared to the general ward.^1–3^ The occurrence of these complications is associated with a poor clinical outcome and a higher risk of mortality.^2^ The majority of these events consist of venous thrombosis and pulmonary embolism.^4,5^ However, arterial ischemic events, such as ischemic stroke have also been described.^6–10^

Possible mechanisms of COVID-19-associated ischemic stroke include generalized coagulopathy, systemic embolism secondary to atrial fibrillation, paradoxical (venous) emboli due to a patent foramen ovale (PFO), arterial thrombosis, and arterial wall inflammation of the cerebral or cervical arteries.^11,12^

Previous studies have mainly investigated symptomatic ischemic stroke. However, it may very well be that “clinically silent” ischemic brain lesions occur due to a procoagulant or proinflammatory COVID-19 response which can impair recovery.^8,13^ Insight into the magnitude, causes and long-term outcomes of cerebral ischemia in hospitalized patients with COVID-19 is crucial for patient care to provide optimal diagnostic strategies and prophylactic and therapeutic treatment.

The CORONIS study was designed to investigate the prevalence, risk factors, and the long-term effects of (silent) cerebral ischemia in hospitalized patients with COVID-19.

## Methods

### Design

The CORONavirus and Ischemic Stroke (CORONIS) study is a multicentre prospective observational cohort study. The study was approved by the medical ethics committee region Arnhem–Nijmegen and all patients will provide written informed consent.

### Patient population

Two hundred hospitalized patients with laboratory-confirmed COVID-19 infection, without a symptomatic ischemic stroke, will be included. In addition, 70 patients with a symptomatic ischemic stroke or transient ischemic attack (TIA) will be included. Ischemic stroke’ must be confirmed with neuroimaging demonstrating either infarction in the corresponding vascular territory or absence of another apparent cause. “TIA” must be diagnosed based on transient focal neurological symptoms lasting <24 h presumed to be due to focal brain, spinal cord, or retinal ischemia without evidence of acute infarction by neuroimaging or pathology (or in the absence of imaging).^14^

The study will be performed in three Dutch academical hospitals: Radboud University Medical Center, Leiden University Medical Center (LUMC), and the University Medical Center Utrecht (UMCU). Patients from other hospitals in the Netherlands can be referred to the participating hospitals for participation in the study. Table 1 summarizes the in- and exclusion criteria.

**Table 1.** Inclusion and exclusion criteria of the CORONIS study.

Inclusion and exclusion criteria:
Inclusion criteria Age ⩾18 years Admitted to the hospital because of COVID-19
Exclusion criteria MRI contraindication and/or post COVID-19 disability interfering with MRI acquisition (e.g. severe delirium) eGFR ⩽ 30 ml/min Pregnancy Limited life expectancy (<3 months) Major disease interfering with study participation or follow-up Not able to give informed consent

COVID-19: coronavirus disease 2019; MRI: magnetic resonance imaging; eGFR: estimated glomerular filtration rate.

### Controls

Age- and sex-matched adult (⩾18 years) controls without previous COVID-19 infection from the general population will be recruited among the patients’ next of kin or social environment.

### Study objectives

The main study objectives are:

1. To determine the prevalence of asymptomatic (silent) cerebral ischemia on MRI in patients with COVID-19 compared to controls.
2. To assess causes of cerebral ischemia in patients with COVID-19.
3. To measure functional outcome and cognitive function in patients with COVID-19 after 3 and 12 months.
4. To determine the incidence of new cerebral ischemia on MRI after 3 months of follow-up in patients with COVID-19.

### Study procedures and follow-up

All eligible patients will be recruited during admission or shortly after discharge. Baseline measurements will be executed during admission or during a visit in the outpatient department (T0). Follow-up at 3 (T1) and 12 (T2) months after inclusion consists of a telephone interview using standardized questionnaires including cognitive assessment. A follow-up brain MRI will be performed 3 months after baseline MRI in a random sample of the patients with COVID-19 (*n* = 120) (T1). In control subjects we will only perform baseline questionnaires and brain MRI. Study procedures are described in Table 2.

**Table 2.** Study assessments.

Assessment	COVID-19 patients			Controls
	Study phase:			
	Baseline (T0)	3 months follow-up (T1)	1 year follow-up (T2)	Baseline
Medical history + vascular risk factors	x	x	x	x
Medication use	x	x	x	x
Recurrent events		x	x	
Demographics	x			x
Questionnaires (education, lifestyle)	x			x
Functional outcome: mRS	x	x	x	x
Functional outcome post-COVID: PCFS		x	x	–
Mood questionnaire: HADS		x	x	–
Cognitive assessment	x	x	x	–
Blood chemistry	x			–
Biobanking	x			–
Contrast transthoracic echocardiography	x			–
48–72 h heart rhythm monitoring	x			–
Brain MRI	x	x		x

mRS: modified Rankin Scale; HADS: hospital and anxiety depression score; PCFS: post-COVID functional scale; MRI: magnetic resonance imaging.

### Baseline questionnaires

#### Demographics, lifestyle, and functional outcome

A structured questionnaire will be used at baseline to assess demographic data (age, sex, body mass index (BMI), ethnicity, and education) and lifestyle behavior. Education will be classified using seven categories: one being less than primary school and seven reflecting an academic degree.^15^ Questions regarding lifestyle include current or past nicotine, alcohol, and illicit drug use. Alcohol consumption is defined as units per day and the age alcohol consumption started (and if applicable stopped). Smoking behavior is defined as the number of pack years, calculated as the number of packs of cigarettes smoked per day multiplied by the number of years a patient has smoked.

Functional performance before hospital admission will be assessed by the modified Rankin Scale (mRS).

#### Medical history

For each patient a history of diabetes mellitus, hypertension, hypercholesterolemia, atrial fibrillation, TIA, ischemic and hemorrhagic stroke, myocardial infarction, peripheral arterial disease, venous thromboembolism, lung diseases, autoimmune diseases, or malignancy will be collected. A past medical history of other neurological disease than the above will be recorded if applicable. The presence of a family history of all of the above, current medication use and vaccination status will be recorded.

### Follow-up questionnaires

Through a telephone interview by one of the researchers, patients will undergo structured questionnaires at 3 and 12 months after baseline testing on the occurrence of new cardiovascular events, lung diseases, and persistence of COVID-19 symptoms such as fatigue and dyspnea. Presence of actual depressive or anxiety symptoms will be assessed using the HADS scale.^16^ Patients will also be asked about current medication use. Functional outcome will be determined using the Post-COVID-19 Functional Status (PCFS) scale and the modified Rankin Scale (mRS).^17–19^

### Brain MRI

Brain MRI scans will be rated qualitatively following a standardized, structured protocol by experienced neuroradiologists blinded to clinical data. The MRI protocol is designed to detect acute and chronic cerebral ischemia, markers of cerebral small vessel disease and vessel wall abnormalities. Table 3 presents the MRI scanning protocol in each participating center.

**Table 3.** Scanning protocol MRI.

		Participating hospital		
		Radboudumc Nijmegen	LUMC	UMCU
Type of scanner		Siemens 3T Prisma	Philips 3T Ingenia	Ingenia Elition 3T X
Contrast agent		15 ml Dotarem^®^ (0.5 mmol/ml)	Clariscan (0.2 ml/kg)	0.1 ml Gadovist/kg
Duration (in min)		40	35	25
T1-weighted	Orientation	3D space fatsat	3D T1	Axial
	Voxels	0.9 mm isotropic	1.15 mm isotropic	0.5 × 0.5 × 0.5 mm
FLAIR	Orientation	3D space flair fatsat	2D FLAIR	Axial
	Voxel + resolution	1 mm isotropic	0.7 × 0.7 × 5.00 mm	0.6 × 0.6 × 4 mm
Diffusion weighted imaging (DWI)	Orientation	Axial Resolve	Axial	Axial
	Target-slice thickness + resolution	5 mm	5 mm	4 mm
Susceptibility weighted imaging (SWI)	Orientation	Axial	3D	Axial
	Target-slice thickness + resolution	3 mm	2 mm	2 mm
Intracranial vessel wall imaging with and without contrast	Orientation	3D space fatsat	3D	Axial
	Target-slice thickness + resolution	0.9 mm isotropic	0.6 × 0.6 × 1.0 mm	0.5 mm isotropic
Diffusion tensor image (DTI)		2 mm B0, 1000, 2000 64 directions	–	–

Radboudumc: Radboud University Medical Center; LUMC: Leiden University Medical Center; UMCU: University Medical Center Utrecht; FLAIR: fluid-attenuated inversion recovery.

#### MRI abnormalities

Brain MRIs will be evaluated for acute or previous ischemic lesions, markers for cerebral small vessel disease, and intracranial vessel wall abnormalities. An acute ischemic lesion is defined by the presence of restricted diffusion on DWI. Markers of cerebral small vessel disease are defined as recent small (sub)cortical infarcts, white matter hyperintensities of presumed vascular origin, and cerebral microbleeds. The markers of small vessel disease are assessed in concordance with the STandards for ReportIng Vascular changes on nEuroimaging (STRIVE) criteria for cerebral small vessel disease.^20^

Intracranial vessel wall abnormalities are defined as major vessel wall changes, such as dissections, occlusions, and stenoses. In addition, images will be assessed for enhancing foci specified for the vessel segment. Vessel wall enhancement is classified as concentric or eccentric type enhancement. The MRI characteristics of interest are showed in Table 4.

**Table 4.** MRI characteristics of interest.

MRI-sequence	Outcome	Additional information
FLAIR	White matter hyperintensities (WMH)	Fazekas (0/1/2/3)
	Previous cerebral infarction	Location: Local, multifocal Cortical, lacunar
	Signs of delayed cerebral hypoxia	
DWI	Acute ischemic lesions, DWI + lesion	Location: Local, multifocal Lacunar, territorial
SWI	Cerebral hemorrhage	Location
	Cerebral microbleeds	Location: lobar, deep Number of lesions: <5, 5–10, >10
T1 intracranial vessel wall imaging	Vasculopathy	
	Mural hematoma (pre-contrast)	Location: MCA, ACA, PCA, BA, or VA Concentric versus eccentric
	Vessel wall abnormalities	Stenosis Dissections Occlusions Enhancement Location: MCA, ACA, PCA, BA, or VA Concentric versus eccentric
T1 post-contrast	Meningeal contrast enhancement	Location: Leptomeningeal/pachymeningeal
	Cranial nerve enhancement	Location
Coincidental findings	Presence/absence	

MCA: middle cerebral artery; ACA: anterior cerebral artery; PCA: posterior cerebral artery; BA: basilar artery; VA: vertebral artery; DWI: diffusion weighted imaging; SWI: susceptibility weighted imaging.

### Cognitive assessment

At baseline, the patients with COVID-19 will undergo a short cognitive screening with the Montreal Cognitive Assessment (MoCA). This 10-min test covers various cognitive domains, including memory, visuoconstruction, attention, executive functioning, and language.^21^ During follow-up, patients will undergo the Telephone Interview for Cognitive Status (TICS), which covers verbal and working memory, orientation, language, and attention.^22^

### Contrast transthoracic echocardiography

To assess presence of PFO, patients will undergo agitated saline contrast transthoracic echocardiography. All echocardiography examinations will be performed by an experienced cardiologist. The interatrial septum will be assessed in multiple views. In addition shunting will be evaluated with color flow Doppler and first-generation contrast. The appearance of microbubbles in the left atrium within three to six cardiac beats after opacification of the right atrium is considered positive for the presence of an intracardiac shunt such as a PFO. Valsalva maneuver will be performed to promote right-to-left shunting of microbubbles to identify a PFO when no shunting is present without provocation.^23^

### Heart rhythm monitoring

Patients will receive an ambulatory Holter to monitor heart rhythm for a period of 48–72 h. Holter monitoring will be performed according to standard procedures. If patients have received rhythm monitoring during admission for standard medical practice (e.g. telemetry during ICU admission ⩾48 h), these data will be used for the current study and no additional Holter monitoring will take place to reduce the burden for the patients.

### Blood sampling

Fifty-four ml blood (18 ml citrated plasma, 20 ml serum, and 10–16 ml EDTA) will be sampled to assess biomarkers of inflammation and coagulation, including genetic variants of these factors. The samples will be stored locally according to the hospital’s regulations or in the affiliated biobank.

### Statistical analysis

#### Sample size calculation

To determine the prevalence of asymptomatic (silent) cerebral ischemia on MRI in patients with COVID-19 compared to controls, we based our sample size calculation on the currently available literature. We expect an incidence of about 1%–3% of symptomatic ischemic stroke in hospitalized patients with COVID-19.^2,6,8,10^ Extrapolating from existing literature we expect a six- to nine-fold increased prevalence of asymptomatic (silent) cerebral ischemia (i.e. 18%–25% assuming a 3% prevalence of symptomatic events) as compared to symptomatic ischemic stroke.^24^ In 200 patients undergoing MRI scanning this would lead to identification of about 40 cases with asymptomatic (silent) cerebral ischemia and at least 160 controls without asymptomatic cerebral ischemia. The observed prevalence will be compared with that in controls. Assuming a prevalence of silent cerebral ischemia in these subjects of max 1%^25^ and of 20% in the patients with COVID-19, we will have 95% power to detect a significant difference at the significance level of 0.05 (95% confidence level).

Regarding objective 2, to assess causes of cerebral ischemia in patients with COVID-19, based on the expected 110 cases with symptomatic (*n* = 70) or asymptomatic (expected *n* = 40) cerebral ischemia and (expected) 160 controls without (a)symptomatic cerebral ischemia we have a power of 95% to demonstrate a relative risk of 3 (alpha 0.05). We will still have 80% power to identify less frequent exposures, for example for exposures with a prevalence of 5% in the controls, we can demonstrate a relative risk of 3.4.

For objective 3 and 4, measuring functional outcome and cognitive function after 3 and 12 months and determining the incidence of new cerebral ischemia on MRI after 3 months of follow-up in patients with COVID-19, the sample size will be equal to that of the population of patients with COVID-19 in the previous sub studies. The precision of the descriptive results for this study will be determined by this study size (no statistical comparisons are made here). A loss to follow-up rate of 10% is taken into account for all the sample size calculations.

#### Analysis of primary outcomes

For objective 1 we will determine the prevalence of silent cerebral ischemia among patients with COVID-19 admitted to or discharged from the hospital and in age and sex matched controls without (previous) COVID-19 infection from the general population including corresponding 95% confidence intervals.

For objective 2, cases, patients with COVID-19 with cerebral ischemia (symptomatic and asymptomatic) and controls (without cerebral ischemia) will be compared with respect to the prevalence of possible risk factors using logistic regression models. Odds ratios will be estimated as measures for the relative risks associated with each possible risk factor. Each risk factor will be analyzed in univariable and multivariable analysis to correct for confounders as age, sex, and comorbidities.

In a follow-up study after 3 and 12 months, we will describe functional performance and cognitive function in COVID-19 patients. We will stratify for subgroups in analysis (symptomatic ischemic stroke, asymptomatic cerebral ischemia, no cerebral ischemia).

After 3 months we will investigate the long-term incidence of asymptomatic (silent) cerebral ischemia in patients with COVID-19. The incidence rate will be determined as the number of new (or first) silent cerebral ischemia divided by the total amount of person-time.

## Discussion

The CORONIS study is a multicentre prospective cohort study investigating the prevalence, risk factors and long-term incidence of (silent) cerebral ischemia in patients hospitalized with COVID-19, and to determine long-term functional outcome.

Little is known about the occurrence and consequences of clinically “silent” cerebral ischemia in patients hospitalized with COVID-19. Research on ischemic stroke as a complication of COVID-19 showed a prevalence ranging from 1% to 3%.^2,6,8,10^ The rate of ischemic stroke as complication of COVID-19 seems to be higher than in other respiratory viruses such as influenza.^7,26^ Recent neuroradiologic studies described multiple brain MRI abnormalities such as (micro)hemorrhage, ischemic lesions, and signs of encephalitis in patients with COVID-19.^27^ Post-mortem pathology studies showed vascular damage, including hypoxic damage, ischemic lesions and (micro)hemorrhages, and inflammatory infiltrates in brain tissue.^28,29^ However, most of our knowledge on these cerebrovascular complications of COVID-19 is derived from retrospective data. Several studies have suggested coagulopathy and endotheliopathy both to be as possible mechanisms of COVID-related ischemic stroke, however no prospective risk factor analysis has been done. To anticipate on the possible consequences of both symptomatic and silent ischemic brain lesions, prospective studies in patients with COVID-19 investigating the effects of COVID-19 in the brain are urgently needed.

Our study is the first to prospectively conduct a brain MRI in hospitalized patients with COVID-19 during the acute phase of their infection. This study will therefore provide more knowledge about the possible effects of COVID-19 on the brain and on cerebrovascular damage in this patient population. Combined with the follow-up MRI we will gain knowledge on dynamics of cerebral ischemia in patients with COVID-19 as well. This can help clinicians to understand mechanisms/causes of COVID-19 related functional loss.

Among the strengths of this study is the multicentre design, leading to a large sample size of patients included from multiple regions throughout the Netherlands. Moreover, the prospective design with two follow-up assessments allows us to collect longitudinal and detailed standardized information, including demographics, vascular risk factors, cognitive tests, and imaging measurements of the patients. Due to our limited exclusion criteria, we will be able to include a patient group with high external validity. Adding matched controls enables us to compare the prevalence of silent cerebral ischemia and other cerebrovascular lesions in both groups of patients.

## Summary and conclusions

In conclusion, CORONIS is a pivotal study to investigate the prevalence, long-term incidence and risk factors of silent cerebral ischemia in hospitalized COVID-19 patients, and will determine long-term functional outcome in this population.
